# Type O blood group associates with higher anti‐JC polyomavirus antibody levels

**DOI:** 10.1002/brb3.2298

**Published:** 2021-07-21

**Authors:** Pia Frenken, Hans‐Peter Hartung, Tomas Olsson, Ortwin Adams, Clemens Warnke

**Affiliations:** ^1^ Institute for Virologie Universitätsklinikum Düsseldorf Düsseldorf Germany; ^2^ Department of Neurology, Medical Faculty Heinrich‐Heine‐University Düsseldorf Germany; ^3^ Clinical Neurosciences Karolinska Institutet Stockholm Sweden; ^4^ Department of Neurology University Hospital of Cologne Cologne Germany

**Keywords:** ABO blood group, natalizumab, PML, polyomavirus, progressive multifocal leukoencephalopathy

## Abstract

**Background:**

Patients with multiple sclerosis (MS) and high anti‐JC polyomavirus (JCPyV) antibodies in blood have an increased risk for the development of progressive multifocal leukoencephalopathy (PML) when treated for MS. To test the hypothesis that type O blood group associates with anti‐JCPyV antibody levels and the risk of developing PML, we characterized ABO blood group antigen on blood samples of 62 patients with PML, and 64 MS controls without PML.

**Methods:**

Monocentric retrospective cohort study. Anti‐JCPyV antibody levels in arbitrary units (AU) were determined in sera using an ELISA‐based method, and blood group specific antibodies using standardised test erythrocytes.

**Results:**

Anti‐JCPyV antibody levels were higher in individuals with blood group O compared with all other blood groups (O: median AU: 129; not O: median AU: 53; p = .005). This association was not observed for the closely related BK virus. Of the 62 patients with PML, 29 (47%, 95% confidence interval (CI) 35%–59%) were of blood group O, which showed a nonsignificant trend to differ from the expected distribution in the German population (41%), and the MS controls studied (36%, 95% CI 25%–48%).

**Conclusion:**

The ABO blood group O antigen associates with higher anti‐JCPyV antibody levels and may impact the risk of the later development of PML. The overrepresentation of blood group O in cases with PML was in line with a previous publication. Larger studies are warranted to assess a potential value of host genetic markers, such as the ABO status, for PML risk prediction during immunotherapy.

## Graphical abstract

Patients with multiple sclerosis and high anti‐JC polyomavirus (JCPyV) antibodies in blood have an increased risk for the development of progressive multifocal leukoencephalopathy (PML) when treated for MS. We tested the hypothesis that type O blood group associates with anti‐JCPyV antibody levels and the risk of developing PML in a monocentric cohort study. Anti‐JCPyV antibody levels were found to be higher in individuals with blood group 0 compared with all other blood groups, and we noted a trend for a higher frequency of blood group 0 in patients with PML.

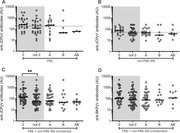

## 1 INTRODUCTION

Progressive multifocal leukoencephalopathy (PML) is an opportunistic infection of the brain caused by JC polyomavirus (JCPyV). PML occurs in patients with impaired cellular immune function such as patients with haematological disorders, patients infected with human immunodeficiency virus (HIV), and it is also a major concern in patients with multiple sclerosis (MS) treated with novel selective immunosuppressive therapies. In particular, the alpha4‐integrin‐blocking medication natalizumab associates with an increased risk of the development of PML (Major et al., 2018), but cases of PML have also been observed with alternative medications such as fingolimod or dimethyl fumarate for the treatment of MS (Warnke et al., 2015), or efalizumab for psoriasis (Schwab et al., 2012), an observation that also hinders the development of several biologicals for immune mediated conditions. Detection of anti‐JCPyV antibodies and the level of the anti‐JCPyV antibody response (so‐called serum index values) (Plavina et al., 2014; Warnke, Ramanujam, et al., 2013) are accepted risk factors for the later development of PML. The reasons why higher antibody levels to JCPyV associate with higher risk of developing PML are not well understood. Host genetic factors may influence the anti‐JCPyV antibody levels, and thus may be the underlying cause for such link. This has been shown for several human leukocyte antigen (HLA) class II variants presenting antigens to CD4+ T cells important in the defense against infections, but studies thus far were also underpowered to display associations outside the HLA region (Sundqvist et al., 2014).

PML is characterized by a lytic infection of oligodendrocytes and astrocytes and is often localized in the subcortical area between the gray and white matter. In this area, there are hemodynamic factors such as the reduction of vessel calibre that might be contributing factors facilitating the evasion of JCPyV. Early studies to detect anti‐JCPyV antibodies were based on the fact that JCPyV can aggregate type O erythrocytes (hemagglutination inhibition assay). Since JCPyV can be present on the surface of B‐lymphocytes, this may induce a clotting of lymphocytes and type O erythrocytes, which could associate with a higher likelihood of viral transmission to the brain in the small arteries in the subcortical area between the gray and the white matter in patients with type O erythrocytes. As such it has been hypothesized that patients with type O erythrocytes may be at higher risk of the later development of PML (Khoury et al., 2013). In this monocentric cohort study, we assessed if type O blood group was associated with higher levels of anti‐JCPyV antibodies.

## 2 METHODS

Stored sera available from routine clinical diagnostics at the Institute for Virology, University of Duesseldorf, collected between January 2000 and July 2016 were used. Sera were selected and grouped based on the results of the JCPyV‐DNA detection in cerebrospinal fluid (CSF) of the same patient, assessed as previously published (Warnke et al., 2011). Individuals who tested positive for JCPyV‐DNA in CSF by qPCR were classified as PML patients. Minimal requirement for the study inclusion was the availability of sufficient sera for blood group studies. Age, sex, and the underlying condition that predisposed for PML were registered with the blood samples as available from the treating physicians.

Individuals with a positive blood/CSF antibody index toward measles, rubella and zoster (so‐called “*MRZ reaction*”) and without the suspicion of PML were classified as non‐PML MS patients (Jarius et al., 2017) and served as control group.

ABO blood types of all patients were determined via “ID‐DiaCell ABO” (Product ID 50520; DiaMed GmbH) reverse grouping meaning determining the blood group via detecting the blood group specific antibodies in the patient's sera (anti‐A, anti‐B) using standardized test erythrocytes (A1, B, O) and patient sera. Sera were tested in an enzyme‐linked immunosorbent assay using a JCPyV‐VP1 protein fused to glutathione S‐transferase (GST) as antigen, and anti‐JCPyV antibody levels and anti‐BKPyV antibody levels in arbitrary units (AU) were determined as previously published (Warnke, Pawlita, et al., 2013; Warnke et al., 2017; Warnke et al., 2014). GraphPad Prism version 8.0 for MAC, GraphPad Software, La Jolla California USA, was used for statistical analysis. Since the serology data were not normally distributed, we used a nonparametric test to compare groups. As we tested the hypothesis that patients with blood group O were at higher risks of PML, we compared the antibody values of blood group O and all other blood groups using the Mann‐Whitney‐U test. Chi‐square test was used to compare data of contingency tables. Results were regarded significant if *p* < .05.

### 2.1 Standard protocol approvals, registrations, and patient consents

The study was approved by the local ethics committee (No. 5683R), waiving the requirement for written informed consent for the retrospective analysis of the stored, anonymized samples at the Institute for Virology, Duesseldorf, for the purpose of this study.

## 3 RESULTS

62 patients with PML and 64 non‐PML MS patients were studied. PML patients were older and non‐PML MS patients had a higher female to male ratio (Table 1). Among the PML patients, the underlying condition causing PML was available in 39 of the 62 patients (63%). The largest group was MS patients treated with natalizumab (27/39, 69.2%), followed by HIV associated PML (7/29, 17.9%) and patients with malignancies (*n* = 5/39, 12.8%).

**TABLE 1:** Patient characteristics and ABO blood groups

	PML	Non‐PML MS^a^
N	62	64
Age (mean, SD)	50.5 (13.4)	45.7 (15.2)
Gender (% female)	45.2	75.0
ABO blood group distribution		
A (n, % (95% CI of %))	23, 37.1% (26.1%–49.6%)	23, 35.9% (25.3%–48.2%)
B (n, % (95% CI of %))	7, 11.3% (5.3%–21.8%)	11, 17.2% (9.7%–28.4%)
AB (n, % (95% CI of %))	3, 4.8% (1.1%–13.8%)	7, 10.9% (5.1%–21.2%)
O (n, % (95% CI of %))	29, 46.8% (34.9%–59.0%)	23, 35.9% (25.3%–48.2%)

Abbreviations: CI, confidence interval; MS, multiple sclerosis; PML, progressive multifocal leukoencephalopathy.

^a^ Non‐PML MS: individuals with no suspicions of PML and a positive MRZ reaction (Jarius et al., 2017).

**FIGURE 1:**
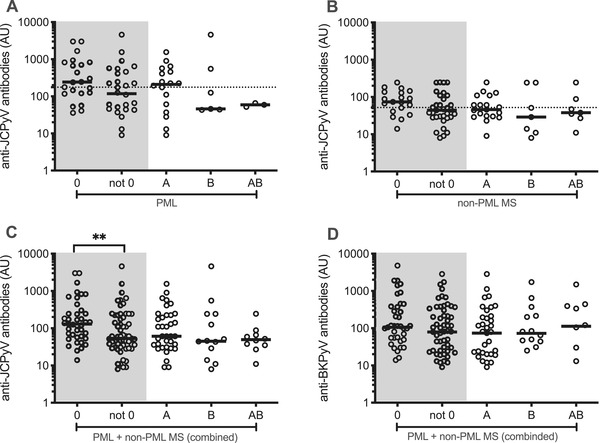
Anti‐JC polyomavirus (JCPyV) antibody levels and the ABO status. Anti‐JCPyV antibodies in arbitrary units (AU) in patients with progressive multifocal leukoencephalopathy (PML) (A), non‐PML multiple sclerosis (MS) patients (B), and combined data (C). Anti‐BKPyV antibodies in AU in patients with PML and non‐PML MS patients (combined data, D). Dotted lines illustrate the median AU values independent of the ABO status in patients with PML and non‐PML MS patients (A and B). **Mann–Whitney *U* test comparing antibody values between blood group O and all other blood groups

The median anti‐JCPyV‐AU values were higher in PML compared with non‐PML MS patients (176 vs. 52, p < .0001). Patients with PML and non‐PML MS patients both displayed a trend for higher median anti‐JCPyV‐AU values if they had blood group O (Figure 1a,b). In the combined dataset of patients with PML and non‐PML MS patients, the difference reached statistical significance (median AU blood group O = 129; median AU all other blood groups: 53, p = .005, Figure 1c). No difference was found for the BKPyV‐AU values in the combined data set (Figure 1d).

Blood group O was overrepresented in patients with PML (PML group: 46.8% vs. MS group: 35.9%, p = .22) (Table 1). In the largest subgroup of patients with PML associated with therapy with natalizumab for MS, a significant difference was observed with blood group type O in 16 of 27 patients (59.3%, 95% CI 40.7%–75.5%, p = .04).

## 4 DISCUSSION

Our study supports the view that, besides HLA associations (Sundqvist et al., 2014), non‐HLA associations may exist that influence the risk of developing PML (Eis et al., 2020; Huang et al., 2017). We here show that anti‐JCPyV serology associates with the ABO blood group classification, with higher anti‐JCPyV antibody levels in individuals of blood group O compared to all other blood types, independently seen in both the PML group and the MS control patients included. It is not understood why patients with MS treated with natalizumab and having higher anti‐JCPyV antibody levels are at increased risk of developing PML. One hypothesis is that higher antibody levels reflect a higher viral replication rate that may facilitate viral genetic mutations, the spread to the brain, and the development of PML. Our findings support this hypothesis. Blood O erythrocytes may have a greater ability to clot with JCPyV carrying lymphocytes in arterioles, and thereby triggering JCPyV transmission to tissue susceptible to asymptomatic (e.g., renal epithelia) or symptomatic brain infection. This could translate in higher anti‐JCPyV immune responses and also higher risk for the development of PML. Fitting this concept, there was a trend for blood group O being overrepresented in patients with PML in our cohort, significant in a subgroup of individuals that developed PML in consequence of natalizumab therapy compared to our MS control group.

PML is a rare and often lethal disease, making prospective controlled studies challenging. We therefore studied samples from the clinical routine, and tested the ABO blood group in these samples. This retrospective monocentric approach leads to limitations inherent to study design, explaining, for example, a predominance of the female gender among MS controls, differences in age, and missing data in particular with regard to the underlying disease in a larger proportion of the PML patients included and outcome data. Furthermore, the MS group had, compared with the general distribution of blood groups in Germany, a lower frequency of blood type O (35.9% vs. the expected 41%; https://www.drk‐blutspende.de/spenderservices/blutgruppen‐und‐verteilung‐in‐der‐bevoelkerung.php, possibly being explained by a bias when using the MRZ reaction status to define this population.

Despite these limitations, our study is in line with a published literature (Khoury et al., 2013), and meta‐analysis of the ABO distribution of the German PML patients included in this study and a US PML population depict a consistent trend with blood group O in PML in 60 of 124 individuals (48.4% (95% CI 39.8%–57.1%)).

Larger host‐genetic studies are underway that associate JCPyV serology with non‐HLA genes to enable a better understanding of the relevance and the mechanisms of the associations found (Huang et al., 2017). Such studies are needed as assessing host genetics with regard to the risk of rare, but severe complications such as PML during immune therapy might pave the way for a more individualized therapy of immune mediated diseases such as MS.

## CONFLICT OF INTEREST

Pia Frenken has nothing to disclose. Hans‐Peter Hartung reports fees for consulting, speaking, and serving on steering committees from Bayer HealthCare, Biogen, GeNeuro, MedImmune, Merck, Novartis, Opexa, Receptos Celgene, Roche, Sanofi‐Genzyme, CSL Behring, Octapharma, and Teva. Tomas Olsson: honoraria, lectures, advisory boards, unrestricted MS research grants, Biogen, Novartis, Roche. Ortwin Adams received speaker's honoraria and research funding from Biogen. Clemens Warnke received institutional support from Novartis, Alexion, Sanofi‐Genzyme, Biogen, Janssen, and Roche.

### PEER REVIEW

The peer review history for this article is available at https://publons.com/publon/10.1002/brb3.2298.

## Data Availability

Anonymized data will be made available by the corresponding author on reasonable request from any qualified investigator.
